# Multicomponent analysis of dietary supplements containing glucosamine and chondroitin: comparative low- and high-field NMR spectroscopic study

**DOI:** 10.1007/s44211-023-00433-2

**Published:** 2023-10-11

**Authors:** Klaudia Adels, Gereon Elbers, Bernd Diehl, Yulia Monakhova

**Affiliations:** 1 Department of Chemistry and Biotechnology, FH Aachen University of Applied Sciences, Heinrich-Mußmann-Straße 1-5, 52428 Jülich, Germany; 2 Spectral Service AG, Emil-Hoffmann-Straße 33, 50996 Cologne, Germany; 3 Institute of Chemistry, Saratov State University, Astrakhanskaya Street 83, 410012 Saratov, Russia

**Keywords:** Glucosamine, Chondroitin sulfate, Polysaccharides, Dietary supplements, High-field NMR, Low-field NMR

## Abstract

With the prevalence of glucosamine- and chondroitin-containing dietary supplements for people with osteoarthritis in the marketplace, it is important to have an accurate and reproducible analytical method for the quantitation of these compounds in finished products. NMR spectroscopic method based both on low- (80 MHz) and high- (500–600 MHz) field NMR instrumentation was established, compared and validated for the determination of chondroitin sulfate and glucosamine in dietary supplements. The proposed method was applied for analysis of 20 different dietary supplements. In the majority of cases, quantification results obtained on the low-field NMR spectrometer are similar to those obtained with high-field 500–600 MHz NMR devices. Validation results in terms of accuracy, precision, reproducibility, limit of detection and recovery demonstrated that the developed method is fit for purpose for the marketed products. The NMR method was extended to the analysis of methylsulfonylmethane, adulterant maltodextrin, acetate and inorganic ions. Low-field NMR can be a quicker and cheaper alternative to more expensive high-field NMR measurements for quality control of the investigated dietary supplements. High-field NMR instrumentation can be more favorable for samples with complex composition due to better resolution, simultaneously giving the possibility of analysis of inorganic species such as potassium and chloride.

**Graphical abstract:**

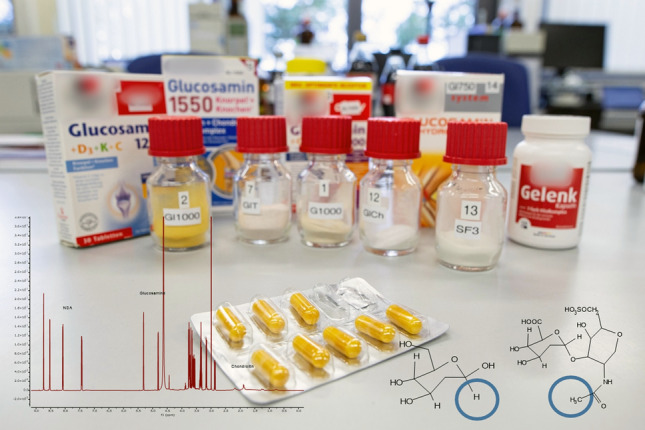

**Supplementary Information:**

The online version contains supplementary material available at 10.1007/s44211-023-00433-2.

## Introduction

Numerous food and dietary supplements have been claimed to reduce symptoms of pain as well as slow disease progression for people with osteoarthritis and are marketed worldwide [1, 2]. Since the year 2000, more than 800 brand name dietary supplement formulations targeting people with osteoarthritis have been introduced, which makes this sector among the most rapidly growing ones in the nutritional supplements and personal care product industry [3]. The most common ingredients in such dietary supplements are glucosamine and chondroitin sulfate (alone or in combination).

With the prevalence of glucosamine- and chondroitin-containing dietary supplements in the marketplace, it is important to have an accurate and reproducible analytical method for the quality control of these compounds in finished products. Currently, high-performance liquid chromatography (HPLC) with ultraviolet (UV) or fluorescence detection is a routine method for the determination of glucosamine in dietary supplements and biological matrices [4–7]. In most of the studies, pre- or post-column derivatization was required to overcome the low sensitivity of glucosamine determination by common HPLC–UV methods because of its weak absorption in the UV range [5–7].

Even more challenging is quantitative analysis of chondroitin sulfate in dietary supplements owing to the wide molecular weight variation, poor UV absorbance, and strong ionic nature of this biopolymer [8]. Therefore, commonly enzymatic hydrolysis of chondroitin sulfate followed by HPLC is used to characterize dietary supplements [9, 10]. Only one direct LC method for chondroitin sulfate determination used octane sulfonic acid in an acidic mobile phase [11]. Unfortunately, this method is not specific in the presence of other components, which do not show interaction with reverse-phase chromatographic column.

To conclude, there is no method of simultaneous determination of all ingredients in dietary supplements aimed at people with osteoarthritis. Moreover, to ensure holistic control of such products, determination of other organic ingredients and inorganic species and possible adulterants is desirable. In this contribution, we focused on the qualitative and quantitative control of such dietary supplements using low- and high-field nuclear magnetic resonance (NMR) spectroscopy. This spectroscopic tool is a unique tool for detection, structural characterization, and quantification of multiple compounds in complex mixtures [12]. However, despite its potential, nowadays NMR is rarely used in routine quality control of dietary supplements. The release of benchtop cryogen-free low-field NMR spectrometers could simplify industrial analysis of these products. The advantages of this technique include no necessity for liquid nitrogen and helium as well as deuterated solvents, low costs and small size.

In this study, NMR methodology was introduced for the simultaneous determination of glucosamine, chondroitin sulfate, methylsulfonylmethane, maltodextrin as well as free organic and inorganic ions, including chloride, potassium and acetate content in dietary supplements. To the best of our knowledge, there is no method for analysis of organic and inorganic composition in this kind of dietary supplements using one instrumental technique. Moreover, this is one of the first applications of low-field qNMR regarding this topic.

## Materials and methods

### Samples and chemicals

In total, 20 dietary supplements in the form of capsules, tablets and granules were investigated (see Table 1 for details). The samples were bought from local drug stores in Germany in June–September 2022. Deuterated water of 99.8% purity containing 0.1% trimethylsilylpropanoic acid (TSP) was obtained from Euriso-top (Saarbrücken, Germany). Sodium salt of ethylenediaminetetraacetic acid (EDTA) was purchased from AppliChem (Munich, Germany). Reference substances D-( +)-glucosamine hydrochloride (> 99%), chondroitin sulfate A/C sodium salt and triethylamine (TEA) were provided by Aldrich (Taufkirchen, Germany). L-Ascorbic acid, phosphoric acid (25%) and KCl were obtained from Roth (Karlsruhe, Germany). Maltodextrin was obtained from Bio Chemika (New Anarkali, Pakistan). N-(9-fluorenylmethoxycarbonyloxy)succinimide (FMOC-succinimide) was purchased from abcr GmbH (Karlsruhe, Germany). Nicotinamide (NSA, purity 99%) and cesium carbonate were purchased from Thermos scientific (Kandel, Germany). Trifluoroacetic acid was provided by CARLO ERBA Reagents GmbH (Emmendingen, Germany).

**Table 1:** Composition of dietary supplements declared by the manufacturers

Sample	Form	Analytes	Matrix ingredients^b^
S1	Capsule	Glucosamine sulfate 500 mg^a^ Chondroitin sulfate 75 mg	Gelatin Magnesium oxide Green-lipped mussel powder
S2	Capsule	Glucosamine sulfate 500 mg Chondroitin sulfate 75 mg	Gelatin Turmeric root extract L-Ascorbic acid
S3	Tablet	Glucosamine hydrochloride 1200 mg	L-Ascorbic acid Polyvinylpyrrolidone Cellulose
S4	Capsule	Glucosamine sulfate–potassium chloride 750 mg Chondroitin sulfate 40 mg	Gelatin Collagen hydrolysate Methylsulfonylmethane
S5	Tablet	Glucosamine sulfate–potassium chloride 775 mg Chondroitin sulfate 50 mg	Cellulose L-Ascorbic acid Polyvinylpyrrolidone
S6	Capsule	Glucosamine sulfate–dipotassium chloride 750 mg Chondroitin sulfate 40 mg	Cellulose L-Ascorbic acid Polyvinylpyrrolidone
S7	Tablet	Glucosamine sulfate 1000 mg Chondroitin sulfate 100 mg	L-Ascorbic acid Cellulose Polyvinylpyrrolidone
S8	Tablet	Glucosamine sulfate–potassium chloride 1500 mg	Cellulose Carboxymethylcellulose Calcium phosphate
S9	Capsule	Glucosamine sulfate 700 mg Chondroitin sulfate 50 mg	Gelatin L-Ascorbic acid Zinc gluconate
S10	Capsule	Chondroitin sulfate 500 mg	Hydroxypropyl methylcellulose Ascorbyl palmitate
S11	Capsule	Glucosamine sulfate 400 mg Chondroitin sulfate 50 mg	Methylsulfonylmethane Gelatin Green-lipped mussel powder
S12	Capsule	Glucosamine sulfate 300 mg Chondroitin sulfate 300 mg	Gelatin Magnesium salts of fatty acids
S13	Tablet	Glucosamine hydrochloride 750 mg Chondroitin sulfate 600 mg	Methylsulfonylmethane Gelatin Green-lipped mussel powder
S14	Tablet	Glucosamine hydrochloride 1200 mg	Cellulose Hyprolose Magnesium stearate
S15	Capsule	Glucosamine hydrochloride 1200 mg Chondroitin sulfate 75 mg	Hydroxy propylmethylcellulose Talcum Silicon dioxide
S16	Capsule	Glucosamine sulfate– dipotassium chloride 500 mg Chondroitin sulfate 375 mg	*Boswellia serrata* extract Ginger rhizome extract L-Ascorbic acid
S17	Capsule	Glucosamine sulfate 156.25 mg Chondroitin sulfate 156.25 mg	Methylsulfonylmethane Gelatin Cellulose
S18	Capsule	Glucosamine sulfate–dipotassium chloride 539.47 mg Chondroitin sulfate 100 mg	Gelatin Methylsulfonylmethane Cellulose
S19	Capsule	Glucosamine sulfate– dipotassium chloride 375 mg Chondroitin sulfate 150 mg	Methylsulfonylmethane Hydroxypropylmethylcellulose L-Ascorbic acid
S20	Granulate	Glucosamine sulfate 1100 mg Chondroitin sulfate 400 mg	Dextrose Collagen hydrolysate Maltodextrin

^a^The weight in mg per capsule tablet declared by the manufacturer

^b^The first three matrix ingredients according to the manufacturer

### Sample preparation for NMR measurements

Cs–EDTA buffer was prepared by weighing of approximately 2.9 g of EDTA and 6 g of Cs_2_CO_3_ and dissolving them in 100 mL D_2_O. Cs^+^ was used as a counterion due to the better solubility of the Cs–EDTA complex in comparison with other cations. The pH was adjusted to 7.0. For quantitative NMR (qNMR) studies, the buffer solution additionally contained 18.75 mg/mL nicotinamide (NSA) as the internal standard for quantification.

For samples in capsules, the shell of ten capsules was opened, emptied, mixed and weighed. For dietary supplements in tablet form, ten tablets with film coatings were finely crushed in a clean porcelain mortar.

80 mg of a sample was weighed and dissolved in 1200 μL of D_2_O or Cs–EDTA buffer for glucosamine or chondroitin sulfate quantification, respectively. The solution was sonicated for 30 min at 50 °C. Then the mixture was placed in the plastic tube and centrifuged (Thermo Scientific, Aachen, Deutschland) at 13,000 rpm (g = 17,000) for 20 min. 600 μL was then transferred in an NMR tube for analysis.

For samples containing paramagnetic compounds/plant extracts, solid-phase extraction (SPE) was additionally performed. SPE cartridges Chromabond® HR-XC (45 μm) were purchased from Macherey–Nagel (Dueren, Germany). Internal standard NSA was added after the purification step to the sample.

Stock solution of KCl (17 mg/mL) was prepared in D_2_O. Subsequent dilutions were made to obtain external calibration solutions in the 0.4–5.3 mg/NMR tube and 0.08–4.9 mg/NMR tube range for K^+^ and Cl^−^, respectively.

### Low-field NMR measurements at 80 MHz

Benchtop NMR measurements at 80 MHz were performed on a Spinsolve 80 Carbon 80 MHz spectrometer equipped with automatic sample changer for 20 samples (Magritek GmbH, Aachen, Germany). ^1^H NMR spectra were recorded with an acquisition time (AQ) of 3.2 s., repetition time (RT) of 30 s., 128 scans (NS), time domain (TD) of 16 K and a pulse angle (PA) of 90°. The data were recorded automatically under the control of Spinsolve software 14.2.1 (Magritek GmbH, Aachen, Germany).

### High-field NMR measurements

High-field NMR measurements were performed on a Bruker Avance III 500 MHz and Bruker Avance NEO 600 MHz spectrometers (Bruker Biospin, Rheinstetten, Germany) with BBFO^PLUS^ Smart and TCI probes, respectively. Both spectrometers were equipped with Bruker Automatic Sample Changer. ^1^H NMR spectra for the samples in D_2_O and in EDTA buffer were recorded with an AQ of 4.5 s., relaxation delay (RD) of 5 s., NS of 16, TD of 65 K and a PA of 30°. The decrease of NS led to considerable decrease in measurement time from 60 min on 80 MHz to 5 min on 500 MHz/600 MHz spectrometers.

Heteronuclei measurements were performed in D_2_O on 500 MHz NMR spectrometer. ^35^Cl NMR spectra were recorded at 90° PA using 1024 NS and four dummy scans (DS). The TD of 4k points were acquired with a spectral width (SW) of 398.4 ppm, AQ of 0.10 s and constant receiver gain (*R* = 362). The following parameters were selected for ^39^K NMR measurements: PA 90°, NS = 128, AQ = 1.10 s, DS = 4, TD = 16k, SW = 2335.0 ppm, RG = 171.7.

### Spectra processing and quantitative analysis

NMR spectra were manually processed using Mestrenova 14.2.3 (Mestrelab Research S.L., Santiago de Compostela, Spain). All spectra were referenced to TSP and then manual phase and baseline correction was performed for the whole spectrum. Line broadening function was set to 0.2 Hz for the ^1^H NMR spectra. Zero filling was set to double of a particular TD value. Integration was performed by summation of all points under a peak (sum integration in Mestrenova) for NSA, glucosamine and ^35^Cl and ^39^K NMR spectra. Due to spectral overlap, peak deconvolution was required for chondroitin sulfate and methylsulfonylmethane (MSM) signals (peak integration mode in Mestrenova). T1 for targeted signals was determined by inversion-recovery experiment. The longest T1 of 4.0 s was determined for NSA.

For the quantification of compounds with the defined structure (α-glucosamine at δ 5.4 ppm, maltodextrin at δ 5.5 ppm, acetate at δ 1.9 ppm, methylsulfonylmethane at δ 3.0 ppm) standard qNMR approach using NSA as the internal standard was used [13]. NSA has four signals between δ 7.0 and δ 9.0 ppm (Fig. 1 in Supplementary information). The signal at δ 7.57 ppm (marked as 1 in Fig. 1 in Supplementary information), which has the smallest T1 time and is not obscured by matrix compounds, was used for qNMR calculations. This signal did not overlap with the signals of matrix compounds in dietary supplements. Due to different non-stoichiometric composition of glucosamine salts (potassium chloride, hydrochloride and sulfate), which are used for the manufacturing of dietary supplements (Table 1), the quantitative results were expressed for glucosamine (C_6_H_13_NO_5_, CAS number 3416–24-8, MW 179.17).

**Fig. 1:**
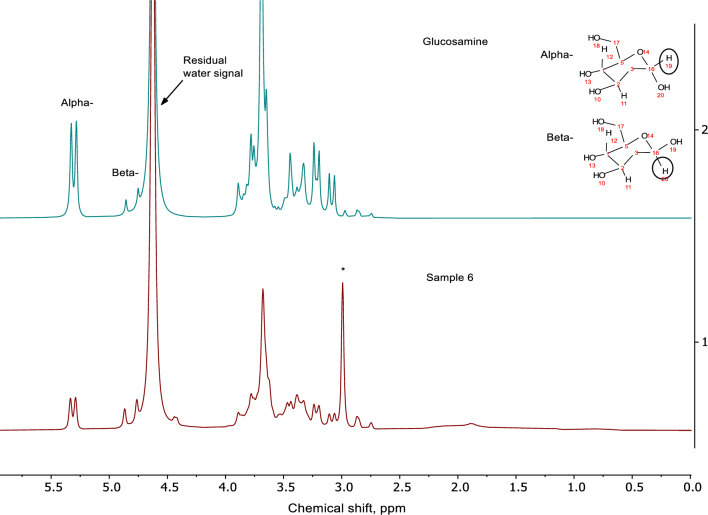
80 MHz NMR spectrum of glucosamine (upper plot) compared with the spectrum of S6 (lower plot). The signal of methylsulfonylmethane (MSM) in S6 is marked by the asterisk

Chondroitin sulfate is a natural biopolymer without a defined molecular weight, which is a prerequisite for standard qNMR calculations. Therefore, alternative quantification routine using a defined mixture of chondroitin sulfate reference standard and NSA was used. For calibration, 20 mg of chondroitin sulfate standard was dissolved in 1200 µL of the Cs–EDTA buffer containing 18.75 mg/mL NSA. Then the integral of the chondroitin signal was adjusted to 100. The resulting NSA integral value (77.5) was used to calibrate the NSA integral value in NMR spectra of dietary supplements, where chondroitin sulfate integral directly showed its percentage relative to the standard. This approach was recently successfully validated for the heparinoid matrix [14].

K^+^ and Cl^−^ were quantified by ^39^K NMR (at δ -0.0 ppm) and ^35^Cl NMR (at δ -3.6 ppm), respectively, by external calibration as reported previously for the heparin matrix [15].

### NMR validation studies

The NMR method was validated for glucosamine and chondroitin sulfate. For reproducibility, three representative samples were prepared, measured, and analyzed five times within 1 day. Moreover, one NMR tube was measured five times in sequence while staying in a magnet. The samples were additionally measured once a day during several days to evaluate the stability of sample solutions. The integral values can be found in Table 1 supplementary information. To evaluate the robustness, several acquisition parameters (NS, AQ, RD, pulse angle) were varied. Limit of detection (LOD) and quantification (LOQ) were determined in matrix as signal-to-noise ratio (SNR) equal to 3 and 9, respectively. The recovery rates were ascertained by adding standard solutions at five concentrations to authentic samples. Accuracy was postulated by the comparison with the results of HPLC reference method for glucosamine.

### HPLC analysis of glucosamine

HPLC analysis was performed on a high-performance liquid chromatograph LC-2010A equipped with an autosampler and UV detector (Shimadzu GmbH, Duisburg, Germany). A Kinetex 6µ EVO C18 100Å (250 × 4.6mm) column was used (KineTeX GmbH, Waldems, Germany).

Quantitative analysis of glucosamine using FMOC-succinimide as derivative agent was adopted without changes regarding sample preparation from [7]. The injection volume was set to 10 µL with a flow rate of 1.0 mL/min at a column temperature of 30°C, detection was performed by recording optical absorption at a wavelength of 265 nm. The sum of the areas of α- (retention time 4.8 min.) and β-isomers (retention time 5.4 min.) was used to quantify the total amount of glucosamine in dietary supplements. The mobile phase gradient program can be found in [7]. The HPLC method based on external calibration was validated in house regarding precision, recovery rate, robustness and LOD/LOQ. Some examples of chromatograms can be found in Fig. 2 in supplementary information. The quantitative HPLC results for all samples are listed in Table 2 in supplementary information.

**Fig. 2:**
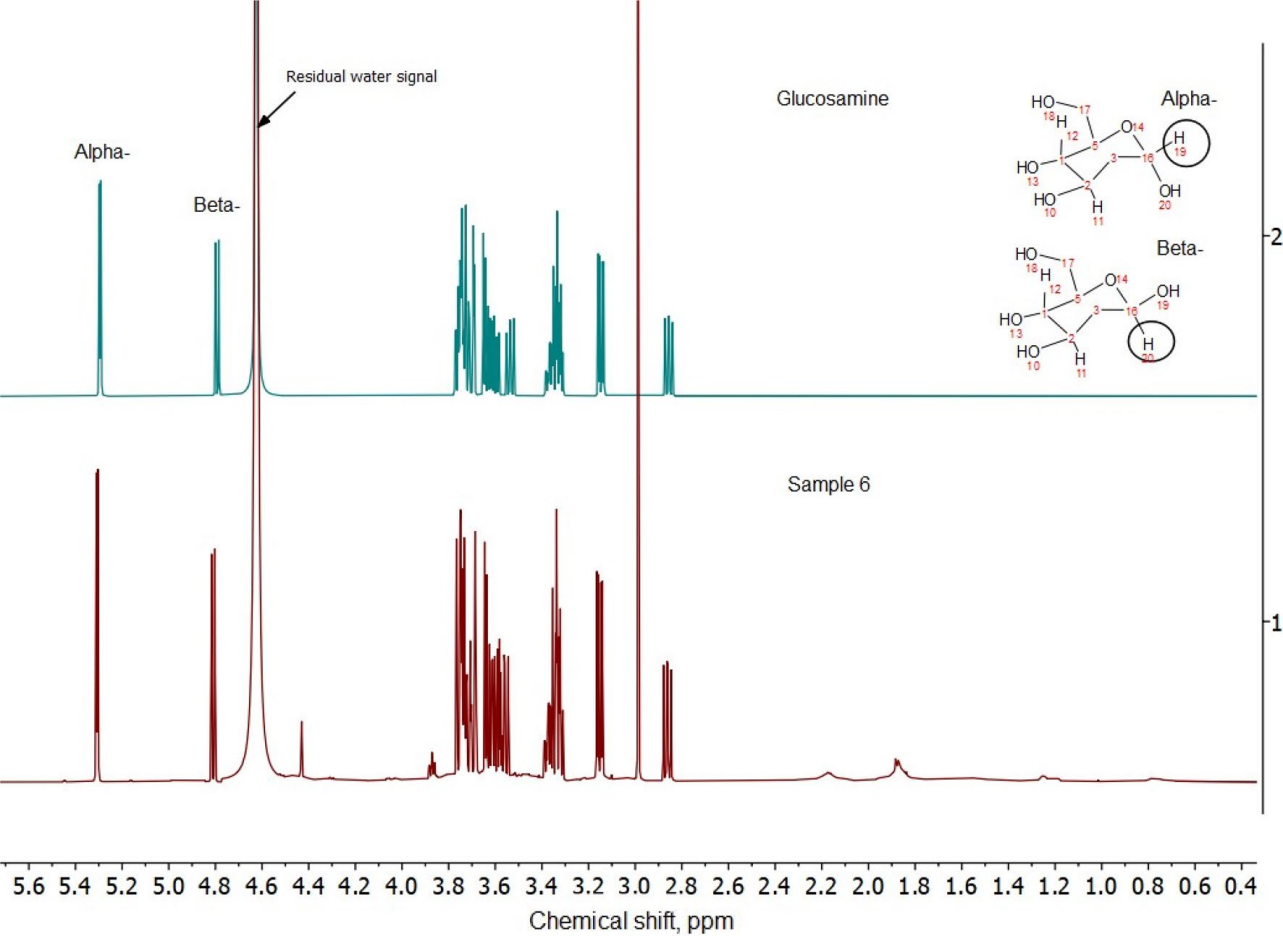
600 MHz NMR spectrum of glucosamine (upper plot) compared with the spectrum of S6 (lower plot)

**Table 2:** Validation results for quantitative analysis of glucosamine and chondroitin sulfate by NMR spectroscopy

Parameter	Glucosamine		Chondroitin sulfate	
	80 MHz	600 MHz	80 MHz	600 MHz
LOD [w/w%]	0.13	0.02	0.25	0.13
LOQ [w/w%]	0.39	0.06	0.75	0.39
Precision [RSD, %] *n* = 5				
Multiple measurements of the same NMR tube	3.4	2.1	3.7	1.8
Multiple sample preparation	4.1	3.5	4.0	2.9
Stability (days)	5		at least 10	
Average recovery [%] *n* = 5	95	96	95	97

## Results and discussion

### Quantification of glucosamine using NMR spectroscopy

NMR spectra of a representative dietary supplement sample compared with glucosamine standard measured at 80 MHz and 600 MHz spectrometers are shown in Figs. 1 and 2, respectively. As expected, resolution on a high-field device was considerably better than on a low-field alternative. The glucosamine signals were grouped into two NMR spectral ranges corresponding to the anomeric (β-anomer at δ 4.8 ppm and α-anomer at δ 5.3 ppm) and the sugar ring protons (δ 2.7–3.8 ppm) (Figs. 1, 2) [16]. As the signal of β-anomer partially overlaps with the water signal, only the signal of α-anomer was integrated for quantification (Figs. 1, 2). The total glucosamine content was then interpolated based on anomeric glucosamine distribution of 51% α‐anomer and 39% β‐anomer as calculated by high-resolution NMR. This is also in accordance with another study [17].

In our preliminary tests, it was found that the resolution was extremely poor for some spectra. In particular, NMR spectroscopy had problems with samples S1, S2, S16 and S20. These samples contained paramagnetic substances such as iron oxide and iron hydroxide (S1 and S2), manganese gluconate (S16) and copper sulfate (S20), which leads to poor shimming and, consequently, to poor resolution of the NMR spectra. Sample S16 additionally contains high amount of plant extracts (see Table 1).

It is known that EDTA can mask paramagnetic ions for NMR measurements. Moreover, bivalent ions such as Ca and Mg, which form stable complexes, can be additionally quantified [15, 18]. Unfortunately, glucosamine oxidized immediately after addition of EDTA buffer. This process led most probably to the formation of glucosaminic acid, which was observable at δ 4.3 ppm [19]. Therefore, to overcome the matrix effect for these four samples, additional sample preparation using SPE cartridges was used. This leads to sufficient resolution for qNMR studies for all problematic samples.

**Fig. 3:**
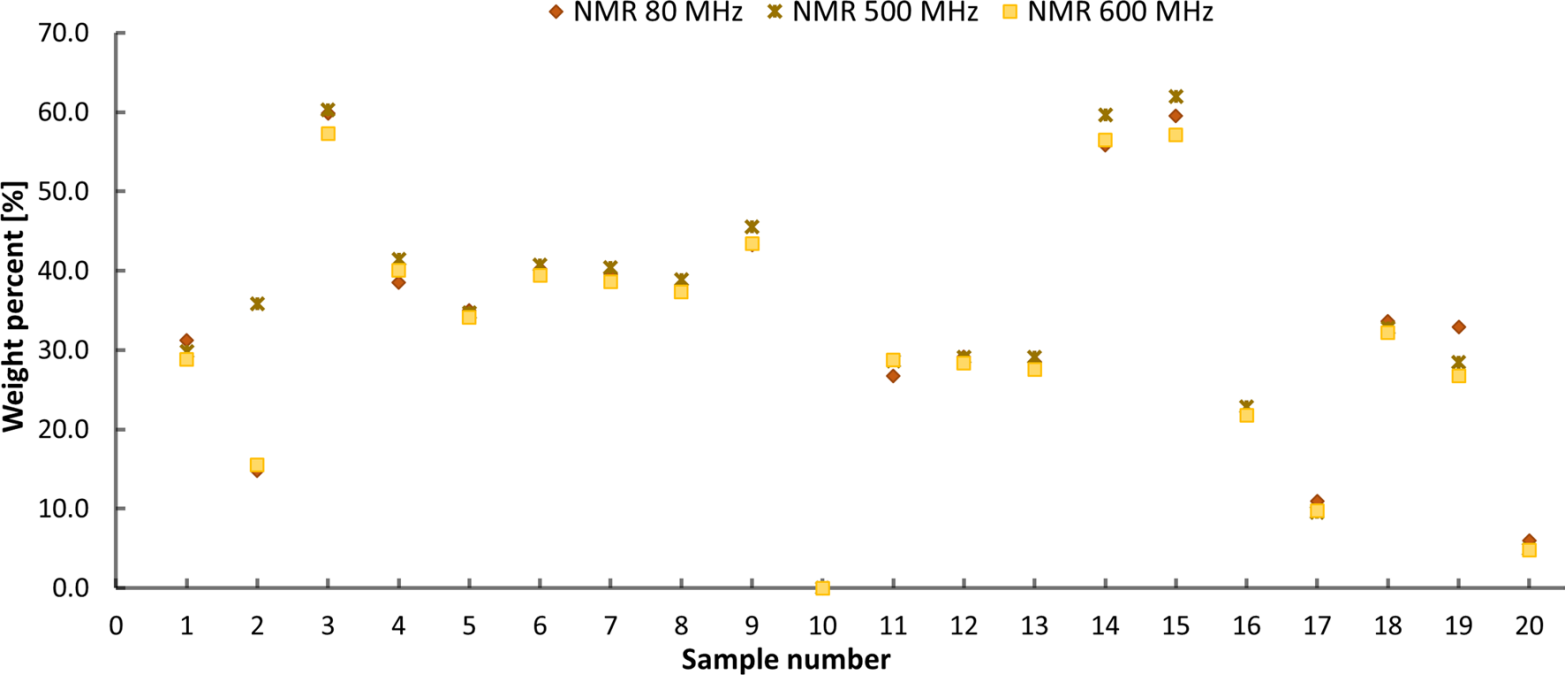
Quantitative NMR results for glucosamine in the investigated samples

The quantitative results for glucosamine by low- and high-field NMR are shown in Fig. 3. and Table 2 in Supplementary information. The glucosamine content obtained by different NMR spectrometers was agreeable within statistical uncertainty (Fig. 3, Table 2 in Supplementary information). Moreover, different high-field NMR devices equipped with NEO console (600 MHz) and room temperature probe (500 MHz) were used to show that the method can be transferred to other NMR spectrometers of different vendors. It was not possible to compare our quantitative results with declared amounts due to non-stoichiometric composition of glucosamine salts used for production of dietary supplements. It can be concluded that low- and high-resolution NMR can be successfully used for quantitative analysis of glucosamine in dietary supplements, provided appropriate sample preparation was used.

### Quantification of chondroitin sulfate using NMR spectroscopy

**Fig. 4:**
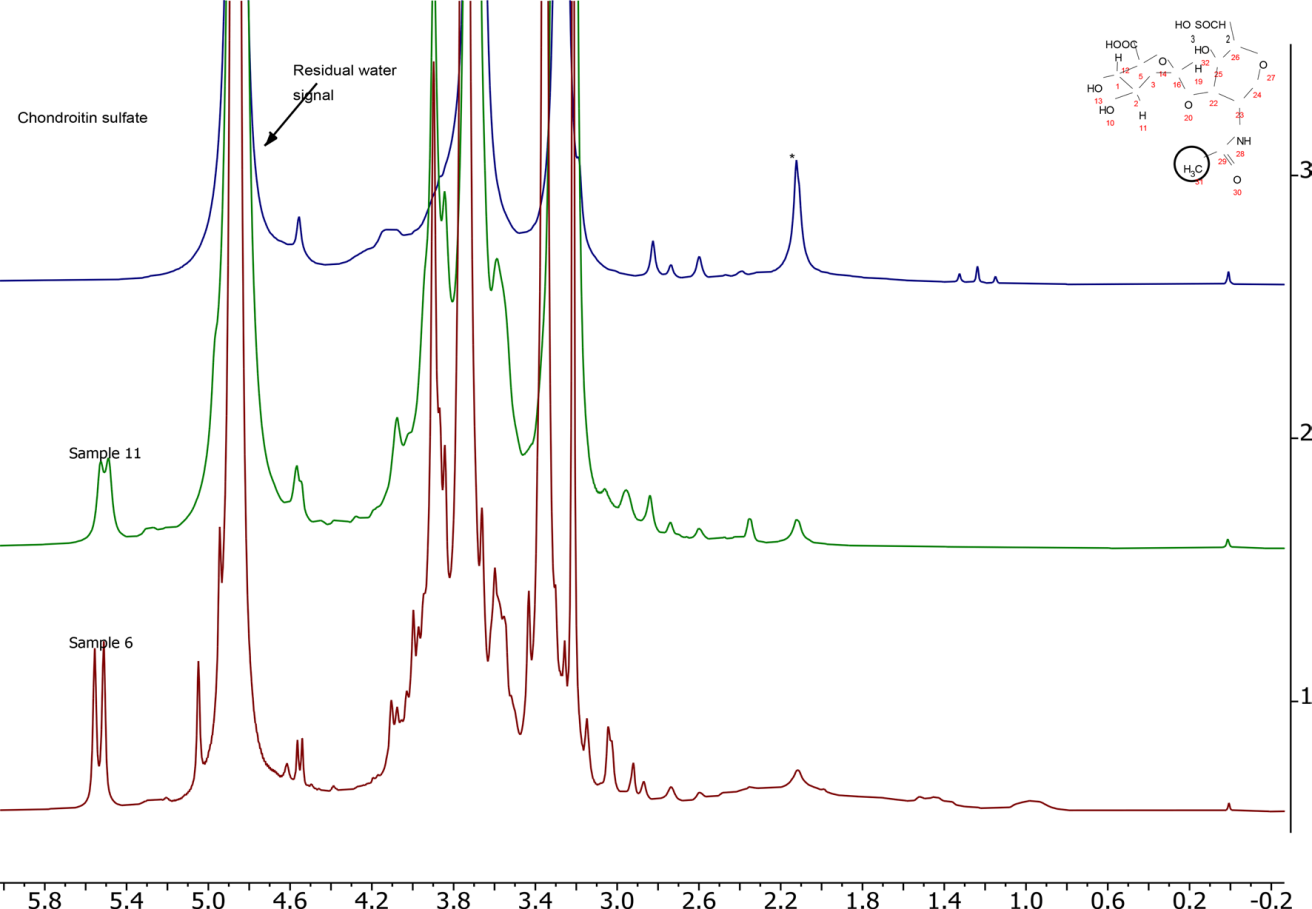
80 MHz NMR spectrum of chondroitin sulfate (upper plot) compared with the spectrum of S11 (middle plot) and S6 (lower plot). The signal of N-acetyl group selected for quantification is marked with an asterisk. Only one repeating unit is shown for the structure of the chondroitin molecule

**Fig. 5:**
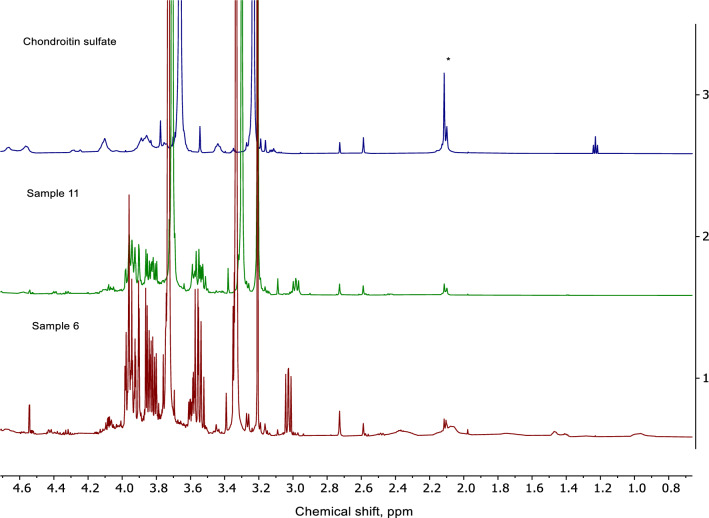
600 MHz NMR spectrum of chondroitin sulfate (upper plot) compared with the spectrum of S11 (middle plot) and S6 (lower plot). The signal of N-acetyl group selected for quantification is marked with an asterisk

^1^H NMR spectroscopy has been already applied for the structure elucidation of chondroitin A/C, especially in the context of studying heparin impurities [14, 20–22]. Figures 4 and 5 show the ^1^H NMR spectra of two dietary supplements in comparison with chondroitin A/C reference standard. The most appropriate signal of N-acetate at δ 2.08 ppm was used for quantification of chondroitin (Figs. 4, 5). Due to addition of EDTA buffer, the NMR spectra of all investigated dietary supplements on both low- and high-field spectrometers were well resolved despite the above-mentioned matrix effect. Therefore, SPE was not performed in this case.

**Fig. 6:**
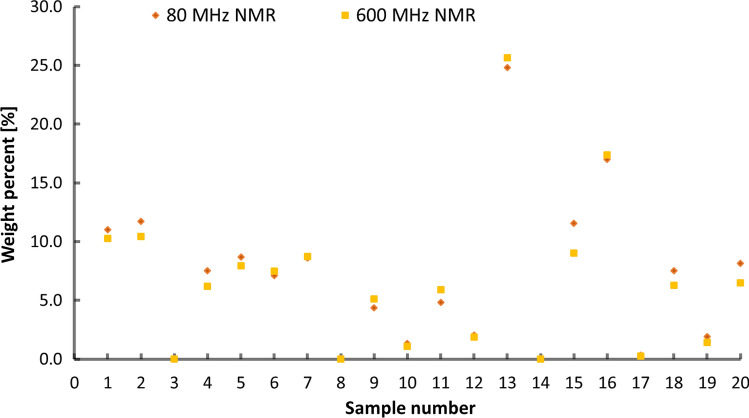
Quantitative NMR results for chondroitin sulfate in the investigated samples

The quantitative results by high- and low-field NMR techniques were comparable for almost all samples (Fig. 6). Moreover, no chondroitin sulfate was detected in S3, S8 and S14 by NMR (Fig. 6). Benchtop NMR overestimated the chondroitin content in the samples S2, S15, S18, and S20 due to poor selectivity in the region of interest. Comparing results with the declared chondroitin amounts, it was found that recoveries above 80% were obtained for 11 of 17 samples, where chondroitin sulfate was labeled by the manufacturer (Table 1). In four samples, less than 5% of the declared amount was found. For two samples, S16 and S18, recoveries of 45% and 57%, respectively, of labeling were obtained.

To conclude, NMR spectroscopy can be used for qualitative and quantitative analysis of chondroitin sulfate in dietary supplements. High-field NMR spectroscopy provided more reliable quantitative results than benchtop NMR due to better resolution.

### NMR method validation for glucosamine and chondroitin

Samples S9 and S11 were selected for validation studies. The NMR method was successfully validated for both analytes, glucosamine and chondroitin sulfate (Table 2). The repeatability of sample measurements achieved standard deviations of < 3.6% and < 4.1% for multiple measurements of the same tube and using repeated sample preparation, respectively. Stability measurements showed that the chondroitin sulfate samples was stable for at least 10 days in EDTA buffer and glucosamine samples in D_2_O for only 5 days due to its oxidation. The limit of detection (LOD) and limit of quantification (LOQ) were defined as the concentration at which the SNR exceeds 3 for LOD and 9 for LOQ in the matrix. The LOD was 0.13% and 0.02% for glucosamine by low- and high-field NMR, respectively. Chondroitin sulfate can be detected starting from 0.25% and 0.13% on 80 MHz and 600 MHz spectrometer, respectively. It should be mentioned that the LOD/LOQ values were related to particular acquisition parameters used for NMR measurements. The values were sufficient to control glucosamine and chondroitin in the investigated products. The average recovery rates were found to be higher than 95% for both analytes.

To estimate the accuracy of glucosamine determination, reference validated HPLC method was used (Table 2 in Supplementary information). No statistical differences between NMR and HPLC results were observed: linear equation had no intercept, slope 1.04 and *R*^2^ = 0.98. Based on these results, NMR spectroscopy seems to be a feasible solution to control glucosamine and chondroitin sulfate in dietary supplements.

### Screening of other constituents by NMR spectroscopy

Apart from glucosamine and chondroitin sulfate, several other compounents can be present studied in dietary supplements. For example, methylsulfonylmethane (MSM) is often used with glucosamine and chondroitin sulfate in dietary supplements [23]. On low- and high-field NMR spectrometers, the resonance of MSM (at δ 3.0 ppm) was well separated and this compound can be directly quantified in aqueous/puffer extracts of the investigated samples (Figs. 1, 2). Quantitative results for MSM analysis in dietary supplements based on low- and high-field NMR data are presented in Table 3. From the analyzed samples, six contained MSM above the detection limit, which is also in accordance with the labeling (Table1). Its content varied between 4.9% (S4) and 32.6% (S17). The labeled and found MSM content were in good agreement with each other (Table 3).

**Table 3:** Quantitative overview of other ingredients in the dietary supplements detected by 600 MHz NMR spectroscopy

Sample	Methylsulfonylmethane			Maltodextrin^a,b^	Acetate^c^	K^+, d^	Cl^−^^, d^
	Labeled	Found					
		80 MHz	600 MHz				
S1	–^e^	−	–	5.0	n.d	1.8	3.7
S2	–	–	–	n.d	0.16	4.8	5.6
S3	–	–	–	n.d	n.d	n.d	n.d
S4	4.9	5.4	5.5	n.d	n.d	5.3	5.2
S5	–	–	–	n.d	n.d	4.6	4.8
S6	4.9	5.0	4.9	n.d	n.d	5.2	4.9
S7	–	–	–	n.d	n.d	5.1	5.3
S8	–	–	–	n.d	n.d	5.0	5.0
S9	–	–	–	n.d	n.d	5.9	7.1
S10	–	–	–	73	0.12	n.d	n.d
S11	26.6	26.8	27.0	n.d	n.d	n.d	3.5
S12	–	–	–	35	0.10	3.9	3.0
S13	5.9	5.8	5.6	n.d	n.d	3.9	n.d
S14	–	–	–	n.d	n.d	7.9	n.d
S15	–	–	–	n.d	n.d	6.5	n.d
S16	–	–	–	n.d	n.d	4.3	4.5
S17	32.6	31.4	31.7	19	0.020	1.6	1.4
S18	–	–	–	n.d	0.018	4.8	4.4
S19	14.3	13.3	12.7	20	n.d	3.3	4.2
S20	–	–	–	14	n.d	n.d	n.d

^a^Average values based on 80 MHz and 600 MHz NMR

^b^Maltodextrin was labeled only in S20

^c^Based on 600 MHz NMR spectroscopy

^d^Based on 500 MHz NMR spectroscopy

^e^Not labeled and not detected by NMR

All contents are expressed in w/w%

Retrospective determination of free acetyl anion in dietary supplements was another goal of this study. The acetate ion could result from the instability of the N-acetylated polysaccharides, for example, chondroitin, and/or could be introduced in the production process. The methyl group of free acetate ions was observed at δ 1.9 ppm in ^1^H NMR spectra and the signal is free from interference from the N-acetyl signal originating from chondroitin sulfate on low- and high-field NMR instruments. Due to the relatively small acetate concentrations, quantitative results were obtained based on 600 MHz NMR data. No free acetate was observed in 16 investigated samples (Table 3). Four samples (S3, S10, S12, S17 and S18) contained acetate anion in small quantities, which does not indicate falsification and/or degradation of chondroitin sulfate.

Maltodextrin is one of the common edible carbohydrates, which is commonly present as inert material in food and dietary supplements [24]. This compound can act as a possible adulterant of dietary supplements, because routine HPLC-based authenticity tests can be deceived by its addition due to poor solubility in widely used organic solvents as well as due to lack of chromophore [23]. Maltodextrin was detected in six investigated dietary supplements at δ 5.5 ppm by both NMR techniques despite being labeled only in S1 (Table 1, 3). Sample S10 contained a large amount of maltodextrin (73%), which, together with a low amount of detected chondroitin sulfate (Fig. 6), could be a sign of possible deliberate adulteration of this product.

Inorganic compounds such as potassium and chloride cannot be determined by the routine ^1^H NMR run. Additional ^39^K and ^35^Cl NMR experiments were used for their detection and quantification using high-field NMR spectrometer. The contents of both ions can be used to unravel the structures of the alleged double/mixed glucosamine salts used for the production of glucosamine-containing dietary supplements (glucosamine hydrocholoride, glucosamine sulfate potassium chloride, glucosamine sulfate dipotassium chloride). Due to these ambiguities, the effective amount of the ingredient could be less than the labeled amount [26].

**Fig. 7:**
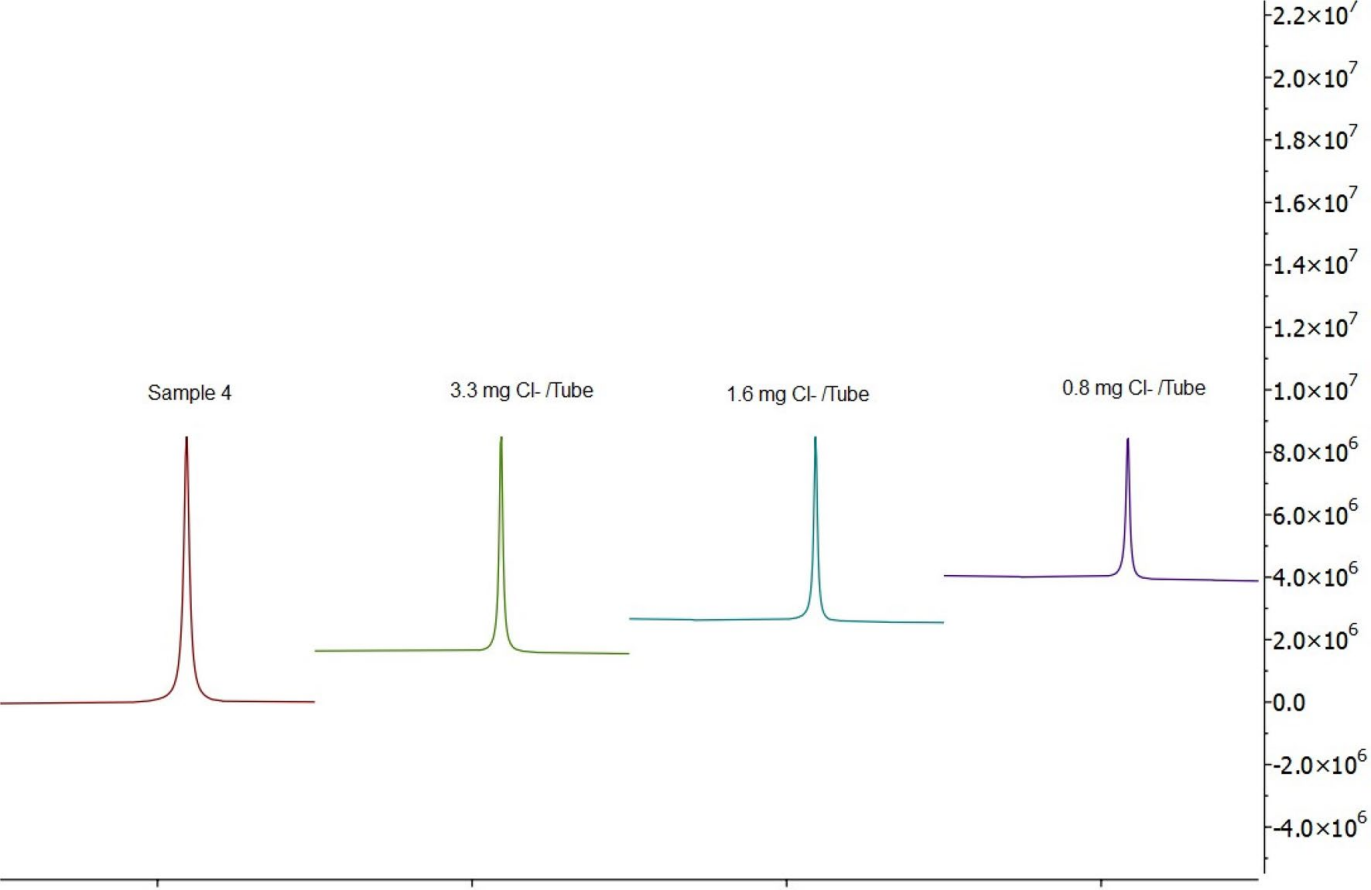
500 MHz ^35^Cl NMR spectra of S4 compared with the KCl standard. The signal has its maximum at δ -3.6 ppm

**Fig. 8:**
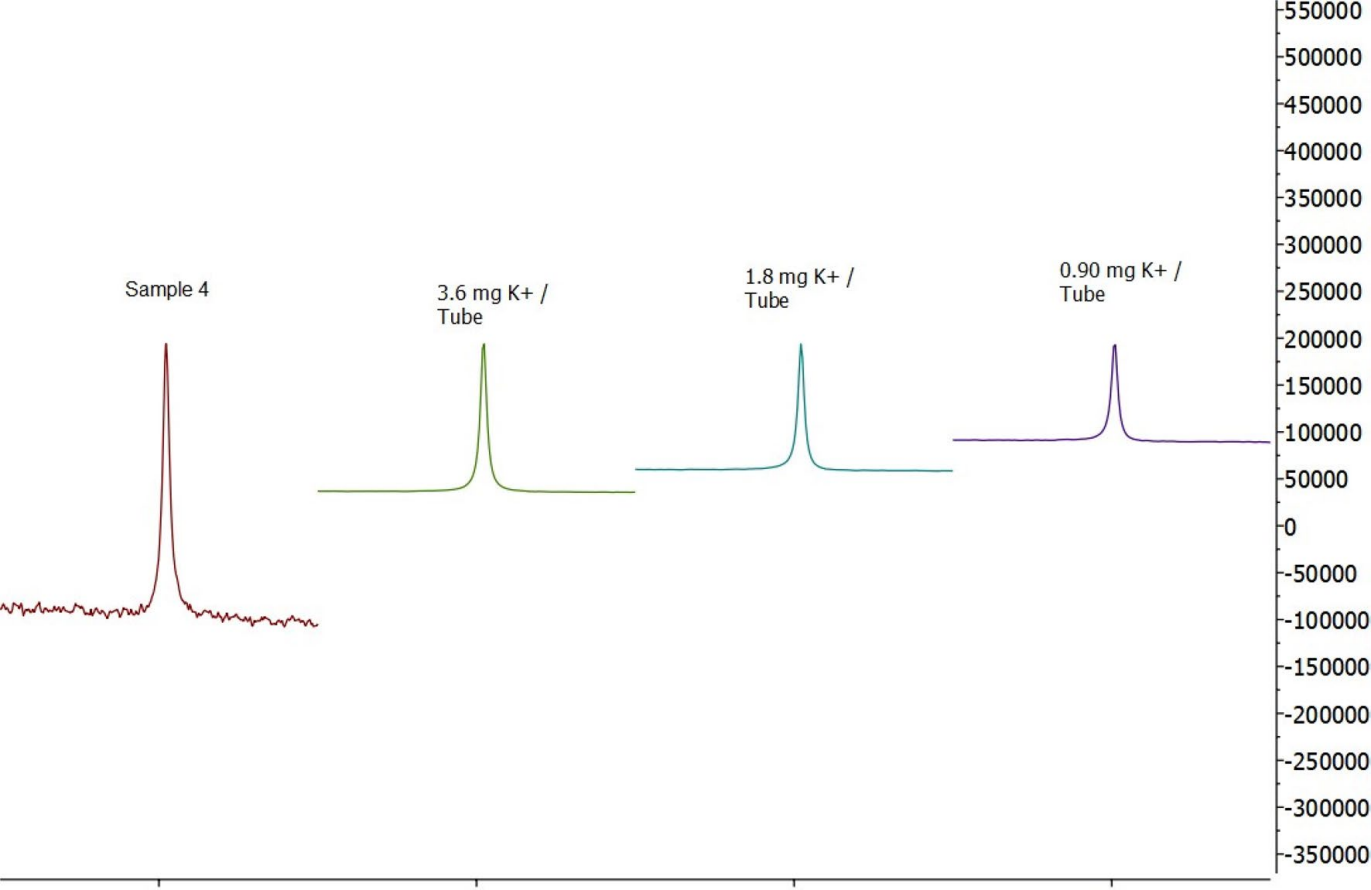
500 MHz ^39^K NMR spectra of S4 compared with the KCl standard. The signal has its maximum at δ 0.0 ppm

Some examples of K^+^ and Cl^−^ NMR peaks originated from KCl standard solutions and the investigated samples are shown in Figs. 7 and 8. The quantification results for K^+^ and Cl^−^ in the investigated samples calculated by the integration of NMR peaks (Figs. 7, 8) are summarized in Table 3. K^+^ and Cl^−^ were detected in 16 and 14 samples, respectively. The data showed K^+^ and Cl^−^ contents with the average values of 4.6 ± 1.6 w/w % and 4.5 ± 1.3 w/w %, respectively. Due to the relatively high content of both ions, we believe that the method can be transferred to other NMR spectrometers equipped with probes less sensitive to the heteronuclei.

Both ions were detected in all samples that were labeled as containing glucosamine sulfate potassium chloride or glucosamine sulfate dipotassium chloride. However, K^+^ was additionally detected in samples with glucosamine sulfate (Tables 1, 3). It means that dietary supplements most probably contained “glucosamine” as physical mixtures of the stable glucosamine chloride with inorganic alkaline salts such as K_2_SO_4_ and KCl. This is also in accordance with the results of synthesis [14]. Therefore, to achieve comparable results, quantitative data were expressed as “glucosamine”. NMR spectroscopy is, therefore, also an appealing tool to quantitatively check inorganic and organic composition of dietary supplements. Our method for the determination of MSM, acetate and maltodextrin does not require additional sample preparation and analytical equipment. High-field NMR was a prerequisite for the quantification of Cl^−^ and K^+^ using the spectrometer pool at our disposal.

## Conclusions

NMR spectroscopy provided an excellent opportunity for multicomponent screening of dietary supplements [27–32]. In recent years, ingredients of several types of dietary supplements including berry-based supplements, curcuma dietary supplements and red yeast rice dietary supplements (DS) were analyzed using high-field NMR [27, 29, 32]. High-field NMR was also a good method to identify adulterants in marketed weight loss supplements, sport nutrition and dietary supplements to increase sexual performance [28, 30, 31].

On the contrary, little is known about the application of compact low-field NMR spectrometers that use permanent magnets for analysis of dietary supplements. Recently, a review was published to highlight different applications of this new technology in combination with chemometrics [33]. In particular, 60 MHz ^1^H NMR spectral data in combination with chemometric modeling was suitable for unveiling medicines as adulterants of slimming dietary supplements [34]. Pages et al. evaluated the potential of a benchtop 60 MHz spectrometry for uncovering adulteration of "100% natural" sexual enhancement and weight loss dietary supplements. It was shown that qNMR led to results similar to those obtained with high-field NMR [35]. However, there were no studies, which investigated the applicability of cheaper benchtop NMR spectroscopy for analysis of dietary supplements using classical qNMR approach.

The results of our studies demonstrated that low-field NMR ensures analytical control of dietary supplements that are claimed to address symptoms of degenerative bone and joint conditions including glucosamine and chondroitin salts, MSM and possible adulterants. High-field NMR is usually more accurate and can be used to support low-field NMR results. Moreover, high-field NMRs can be additionally used to quantify inorganic ions (preliminary K^+^ and Cl^−^, but also F^−^, Na^+^, Mg^2+^, Ca^2+^ and others on demand).

### Supplementary Information

Below is the link to the electronic supplementary material.

Supplementary file1 (DOCX 138 KB)

## Data Availability

The datasets generated during and/or analyzed during the current study are available from the corresponding author on reasonable request.
