# Supplementary material for: Multicomponent analysis of dietary supplements containing glucosamine and chondroitin: comparative low- and high-field NMR spectroscopic study
Source: Anal Sci. 2023 Oct 11;40(1):101–13. doi: 10.1007/s44211-023-00433-2 (PMC10766784; doi:10.1007/s44211-023-00433-2)
Supplement: Supplementary file 1 — Supplementary file1 (DOCX 138 KB) [file 44211_2023_433_MOESM1_ESM.docx]

**Fig. 1.** NMR spectrum of the sample S6 with NSA recorded at 600 MHz (upper plot) and 80 MHz (lower plot). The signals of NSA are marked 1 to 4 and have the following T1 times: 4.0 sec. (1), 5.3 sec. (2), 8.1 sec. (3), 6.4 sec.(4).


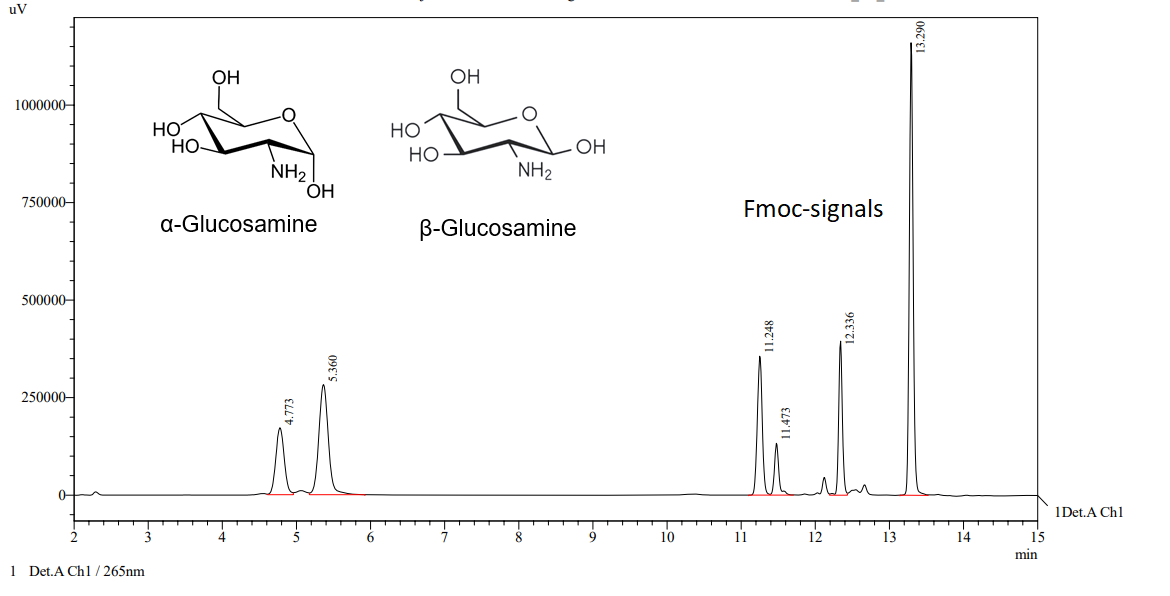


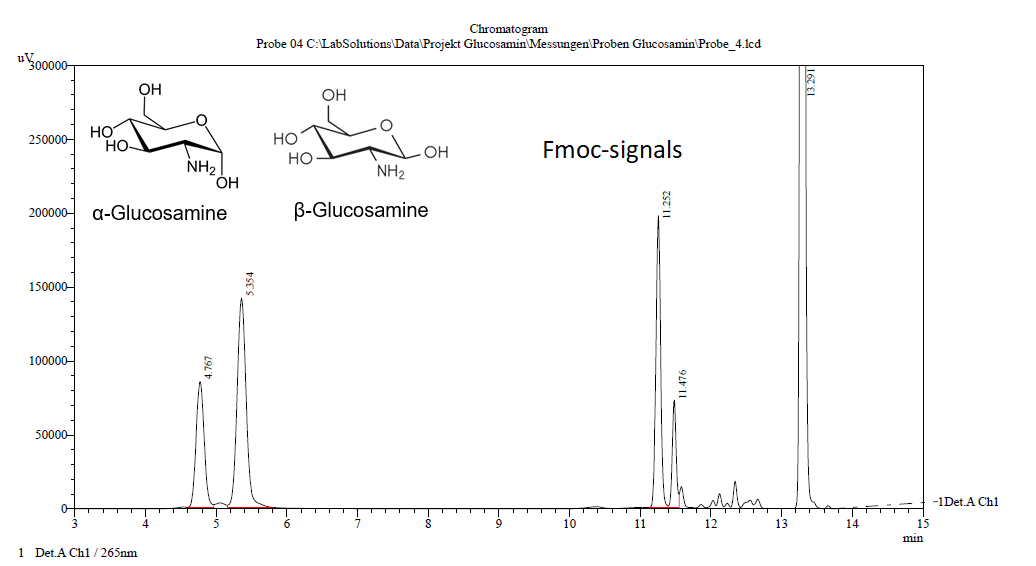


**Fig.2.** Chromatograms obtained for glucosamine standard 80 mg/L (upper plot) and for the S4 with glucosamine content 37.2% using FMOC as derivative agent. Details of experimental conditions are described in Materials and Methods section and in [7].

**Table 1.** Integral values for glucosamine, chondroitin sulfate and NSA for precision calculations.

| Measurement (one sample) | Integral (α-glucosamine) at 80 MHz | Integral (α-glucosamine) at 600 MHz | Integral (NSA) |
| --- | --- | --- | --- |
| 1 | 0.674 | 0.691 | 1.00 |
| 2 | 0.704 | 0.690 | 1.00 |
| 3 | 0.693 | 0.704 | 1.00 |
| 4 | 0.688 | 0.695 | 1.00 |
| 5 | 0.737 | 0.725 | 1.00 |
| CV [%] | 3.4 | 2.1 |  |
| Measurement (different sample preparation) |  |  |  |
| 1 | 0.751 | 0.700 | 1.00 |
| 2 | 0.695 | 0.741 | 1.00 |
| 3 | 0.751 | 0.702 | 1.00 |
| 4 | 0.695 | 0.752 | 1.00 |
| 5 | 0.702 | 0.702 | 1.00 |
| CV [%] | 4.1 | 3.5 |  |
|  |  |  |  |
| Measurement (one sample) | Integral (Chondroitin) at 80 MHz | Integral (Chondroitin) at 600 MHz | Integral (NSA) |
| 1 | 0.135 | 0.128 | 1.49 |
| 2 | 0.131 | 0.129 | 1.49 |
| 3 | 0.130 | 0.131 | 1.49 |
| 4 | 0.132 | 0.125 | 1.49 |
| 5 | 0.122 | 0.130 | 1.49 |
| CV [%] | 3.7 | 1.8 |  |
| Measurement (different sample preparation) |  |  |  |
| 1 | 0.131 | 0.139 | 1.49 |
| 2 | 0.140 | 0.134 | 1.49 |
| 3 | 0.131 | 0.139 | 1.49 |
| 4 | 0.141 | 0.138 | 1.49 |
| 5 | 0.130 | 0.130 | 1.49 |
| CV [%] | 4.0 | 2.9 |  |

**Table 2.** Quantitative NMR and HPLC results for glucosamine in the investigated samples

| **Sample** | **HPLC** | **NMR 80 MHz** | **NMR 500 MHz** | **NMR 600 MHz** |
| --- | --- | --- | --- | --- |
| 1 | 33.9 | 31.2 | 29.8 | 28.8 |
| 2 | 35.6 | 34.8 | 35.8 | 35.6 |
| 3 | 56.4 | 59.8 | 60.3 | 57.3 |
| 4 | 37.2 | 38.5 | 41.5 | 40.1 |
| 5 | 32.3 | 35.0 | 34.7 | 34.1 |
| 6 | 39.9 | 40.20 | 40.7 | 39.4 |
| 7 | 37.7 | 39.53 | 40.4 | 38.6 |
| 8 | 36.6 | 37.67 | 38.9 | 37.3 |
| 9 | 43.4 | 43.23 | 45.6 | 43.5 |
| 10 | Not detected | | | |
| 11 | 30.1 | 26.76 | 28.6 | 28.8 |
| 12 | 25.8 | 29.16 | 29.1 | 28.3 |
| 13 | 27.6 | 28.49 | 29.1 | 27.5 |
| 14 | 53.5 | 55.86 | 59.7 | 56.5 |
| 15 | 61.8 | 59.53 | 62.0 | 61.0 |
| 16 | 20.4 | 21.8 | 22.9 | 21.8 |
| 17 | 10.1 | 10.93 | 9.50 | 9.76 |
| 18 | 32.8 | 33.63 | 32.8 | 32.2 |
| 19 | 30.1 | 32.92 | 28.4 | 26.7 |
| 20 | 5.27 | 5.95 | 4.90 | 4.80 |
